# Supplementary material for: Learning curve and surgical outcome of robotic assisted colorectal surgery with ERAS program
Source: Sci Rep. 2022 Nov 29;12:20566. doi: 10.1038/s41598-022-24665-w (PMC9709162; doi:10.1038/s41598-022-24665-w)
Supplement: Supplementary file 1 — Supplementary Information 1. [file 41598_2022_24665_MOESM1_ESM.zip › LSC Raw data-Ñ[▒K/146/H_ñw╝╨░O▒Kñσ.pdf]

正式摘要(電子簽章：000000233058190)

W52 / W52 ☐英文標題(注意:無英文姓名-請補病人基本檔!) ☐【讀取】憑證

臺中榮民總醫院

出院病歷摘要

轉入號： 轉出號：

[redacted]

(8)流水編號	
(9)入院日期	110/12/15 (2021) 直腸外科 W52 - 081
(10)轉科日期	
(11)出院日期	110/12/22 (2021) 住院天數計 7 日

診斷

- 入院 : 1. S-colon diverticulitis or cancer
  2. s/p Catheter ablation several years ago
- 出院 : 1. S-colon diverticulitis or cancer, s/p Laparoscopic AR on 2021/12/16
  2. s/p Catheter ablation several years ago

主訴

- Abdominal LLQ pain for months

病史

- This is a 43 years old male patient with history of
  1. s/p Catheter ablation several years ago
  According to the medical record, the pateint suffered from left lower abdominal pain several times and last abdominal CT done on 2021/11/06 at LMD showed S-colon diverticulitis. He visited our CRS OPD for second opinion due to persisted symptoms. We arranged abdominal CT on 2021/12/01 for him and the RAD reported that S-colon cancer can't be rule out. After discussion with the pateint, this time he was admitted to our ward for Laparoscopic AR.

體檢發現

-
  - General appearance :
    - easy looking , conscious clear , without cardiopulmonary distress
    - Vital signs口動帶入date 體溫(BT) 脈搏(P) 呼吸(R) 血壓(BP) 身高(BH) 體重(BW) BMI
      20211215 36.3℃ 81次/分 19次/分 170/121mmHg 172.3cm 75.8kg 25.5
    - Vital signs: B.P. 170/121 mmHg , P.R. 81 /min , R.R. 19 /min , B.T. 36.3 ℃
  - HEENT :
    - Eyes: no anemic conjunctiva, no icteric sclera, normal EOM
    - ENT: no otorrhea, no rhinorrhea, no erythematous mucosa, no injected tonsil, no deformity
  - Neck: supple, freely movable, trachea midline,no deformity, no engorged jugular vein , no palpable thyroid or lymph node enlargement
  - Chest:
    - Inspection: Grossly normal, symmetric and fully expansion, no accessory muscle used, no operation scar, no spider angiomata, no gynecomastia
    - Palpation: no palpable mass or nodule, no subcutaneous emphysema, no tenderness
    - Ausculation: clear breathing sound
  - Heart:
    - Percussion: normal heart size
    - Ausculation: regular heart beat, normal S1, S2, no gallop, no murmur
  - Abdomen:
    - Inspection: in
      - flat contour, symetrically, no scar, no stria, no dilated veins, no rashes
    - Ausculation: normoactive bowel sound, no abdominal bruits
    - palpation: soft, no muscle guarding or rigidity, no local tenderness, no rebounding pain, no palpable mass, no palpable pulsation
    - Percussion: no shifting dulllness, no hepatomegaly, no splenomagaly
  -

Peripheral pulsation :	CA	BA	RA	FA	PA	PTA	DPA
Right side :	++	++	++	++	++	++	++/+++
Left side :	++	++	++	++	++	++	++/+++

  - Genito-anus: no tenderness or palpable mass lesion or rectal shelf, normal external genitalia, normal anal tone, brown stool

CRS CONDITION

-
  - Lung

手術日期及方法

- 手術日期及方法20211216 1. Laparoscopic anterior resection
  2. Intraoperative colonoscopy : (-)
  3. High ligation (+) : At IMA root
  4. EndoGIA (60-4.1) X1 + EEA (29 mm) were applied
  5. Proximal cut end: 3cm, distal cut end: > 10 cm
  6. No drainage tube was placed into the abdominal cavity
  7. The ADHESION BARRIER FILM was then placed below the para-umbilical extended wound.
  8. The colon was not taken down from the splenic flexure site
- 手術結果及發現 :
  - 手術日期: 1101216 麻醉方式:
    手術名稱:1. Laparoscopic anterior resection 2. Intraoperative colonoscopy : (-) 3. High ligation (+) : At IMA root 4. EndoGIA (60-4.1) X1 + EEA (29 mm) were applied 5. Proximal cut end: 3cm, distal cut end: > 10 cm 6. No drainage tube was placed into the abdominal cavity 7. The ADHESION BARRIER FILM was then placed below the para-umbilical extended wound. 8. The colon was not taken down from the splenic flexure site
    術前診斷:Diverticulosis and diverticulitis or Ca of S colon
    術後診斷: Diverticulitis s/p laparoscopic anterior resection

住院治療經過

- After admission, he received Laparoscopic AR on 2021/12/16. He was under ERAS protocol, removed Foley catheter on 12/17. He could tolerate soft diet on 12/20, and the abdominal pain gradually improved. Under relative stable condition, he discharged on 12/22, OPD follow up and pending pathology report.

合併症

一般檢查

- 累積報告 :

DATE	TIME	ALB	TP	BIL.T	BIL.D	ALKP	AST	ALT	LDH	AMY	LIPASE	NH3	Ferritin	UA
1101201	1351					85		17		56				
1101208	1111	4.5		0.4	0.1	104	18	19	176					
1101215	1853	4.4	7.3	0.4	0.1	90	23	20						9.4
1101216	2127	3.8		0.67				18						

DATE	TIME	WBC	RBC	HGB	HCT	MCV	PLT	BLST	NEUT	BAND	SEG	LYM	MON	EOS	BAS

1101201	1352	7090		16.2			238		73.1			22.3	3.2	0.8	0.6
1101208	1111	6380	5.39	15.7	47.5	88.1	203		62.0			29.0	5.2	3.0	0.8
1101215	1852	6450	5.22	15.4	46.4	88.9	226		53.2			39.1	4.2	2.9	0.6
1101216	2127	10170		14.7	45.1		195		92.5			6.7	0.8	0.0	0.0

DATE	TIME	NA	K	CL	BUN	CREAT	eGFR	CA	Mg	P	CRP	ESR	PCT	D-Dimer	LACTATE
1101201	1351	137	3.8		16	1.49	54.71				0.159				
1101201	1352														11.9
1101208	1111	142	4.3		19	1.20		9.3							
1101215	1853				16	1.43	57.37				0.272				
1101216	2127	140	4.0		11	1.26		8.6							

DATE	TIME	GLUCOSE	HbA1C	CHOL	TG	HDL	LDL	KETONE	CK	CKMB	TRO-T	PT-P	APTT-P	NT-pro BNP
1101201	1351	94							109	16	6.92			
1101208	1111	83										10.3	28.1	
1101215	1851	90												
1101215	1853			186	246	50	99							

DATE	TIME	PH	BEB	SCVO2	PO2	HCO3	HGB	PCO2	TCO2
1101201	1352	7.390	-2.9	94.4	70.8	21.4		36.1	22.5

- 放射線報告 :檢驗項目 **:CT,COLON WITH AIR INFLATION** 報告日期 **:1101202**

上 訴：
Result:Y
Finding:
Imaging Report Form for Colorectal Carcinoma (AJCC 8th)
1. Imaging Date: 20211201
Imaging Modality, Imaging by
CT scan
Imaging Protocol: CT of colon with air inflation and IV contrast enhancement:
2. Tumor location / Size
Location:
Sigmoid
Size:
Measurable: 6-10cm cm (largest diameter)
3. Tumor invasion
Tumor within colonic or rectal wall
4. Regional nodal metastasis
No
5. Distant metastasis (In this study)
No or Equivocal
6. Other findings: > A case of colon cancer screening.
> Symmetric wall-thickening of the anus, suspect hemorrhoids.
> Diverticulosis over S-colon, D-colon and splenic flexure.
> Irregular wall-thickening of S-colon(6-10cm) with focal stricture and adjacent mesenteric fat stranding (Ser302#74). Colon cancer is highly suspected. Recommend biopsy.
> A 9mm sessile polypoid lesion at splenic flexure of colon(Ser302#23), and a small polypoid lesion about 3 mm in size in D-colon(Ser301#44). Colon polyps are suspected.
> Mild fatty liver.
> A low attenuation lesions over the S2 of liver, in favor of cysts.
> Right tiny renal cyst less than 0.8cm.
> No abnormal focal lesion in the gallbladder, pancreas, spleen, left kidney and bilateral lung fields.
> No significant lymphadenopathy in the abdomen or retroperitoneum.
Impression:
1. Segmental irregular wall-thickening of S-colon with focal stricture and adjacent mesenteric fat stranding. Colon cancer is highly suspected. Recommend biopsy.
2. A 9mm sessile polypoid lesion at splenic flexure of colon and a small polypoid lesion about 3 mm in size in D-colon, suspect colon polyps.
2. Diverticulosis over S-colon, D-colon and splenic flexure.
3. Symmetric wall-thickening of the anus, suspect hemorrhoids.
For other details, please see the above descriptions.

特殊檢查

病理報告

其它

出院時情況

- 3.改門診治療

出院指示

藥名	劑量	劑量單位	用法	頻次	數量	數量單位	現況	天數	單位
Norvasc tab 5mg	1	TAB	PO	QD	7	TAB	TH	7	天
(I I)Takepron OD tab 30mg	1	TAB	PO	QDAC	7	TAB	TH	7	天
Mag. Oxide tab	2	TAB	PO	TID	42	TAB	TH	7	天
OCAS Hamalidge 0.4 mg	1	TAB	PO	QDAC	7	TAB	TH	7	天
Ultracet tab	1	TAB	PO	BIDPRN	14	TAB	TH	7	天

出院計劃

- 門診日期
  - date on 2022/01/05

主治醫師_2062F 蔣鋅帆_______ / 住院醫師_2339C 蔡濬樟_______

正式摘要(電子簽章：000000233058190)

W52 / W52

doctorRank:B
